# Adjunctive Sitagliptin Therapy in Postoperative Cardiac Surgery Patients: A Pilot Study

**DOI:** 10.1155/2012/810926

**Published:** 2012-09-27

**Authors:** Marcia L. Brackbill, Ateequr Rahman, Jeffrey S. Sandy, M. Denton Stam, Arthur F. Harralson

**Affiliations:** ^1^Winchester Medical Center, Heart and Vascular Center, Winchester, VA 22601, USA; ^2^Department of Pharmacy Practice, Bernard J. Dunn School of Pharmacy, Shenandoah University, 1775 North Sector Court, Winchester, VA 22601, USA

## Abstract

*Aim*. We aimed to determine if sitagliptin added to standard postoperative standardized sliding-scale insulin regimens improved blood glucose. *Methods*. A prospective, randomized, double-blind, placebo-controlled pilot study was conducted in diabetic cardiac surgery patients. Patients received sitagliptin or placebo after surgery for 4 days. The primary endpoint was to estimate the effect of adjunctive sitagliptin versus placebo on overall mean blood glucose in the 4-day period after surgery. *Results*. Sixty-two patients participated. Repeated measures tests indicated no significant difference between the groups in the overall mean blood glucose level with a mean of 147.2 ± 4.8 mg/dL and 153.0 ± 4.6 mg/dL for the test and the control group, respectively (*P* = 0.388). *Conclusions*. Sitagliptin added to normal postoperative glucose management practices did not improve overall mean blood glucose control in diabetic patients in the postoperative setting.

## 1. Introduction

Glucagon-like peptide-1 (GLP-1) augments glucose-stimulated insulin secretion in the pancreas and limits new glucose production in the liver [1]. GLP-1, however, is rapidly degraded by dipeptidyl-peptidase IV (DPP-4) [2, 3]. Sitagliptin is an oral medication indicated for type 2 diabetes mellitus (T2DM) and prevents inactivation of GLP-1 by selectively inhibiting DPP-4, thus increasing insulin secretion and decreasing glucagon concentration in a glucose-dependent manner [1, 4]. Sitagliptin has a lower incidence of hypoglycemia compared to other oral antidiabetic agents [5, 6]. The only adverse drug events (ADE) associated with sitagliptin occurring in greater than 5% of patients in clinical trials were headache, nasopharyngitis, and upper respiratory tract infection [4].

To date, no information exists regarding experience with sitagliptin in the acute care setting. GLP-1 infusions have been evaluated for glycemic control in patients undergoing cardiac surgery and have been found to provide better glycemic control than standard therapy [7]. However, GLP-1 is rapidly degraded, and therapy requires a continuous infusion [8]. Sitagliptin is an attractive agent to investigate due to the lower incidence of hypoglycemia compared to other oral agents and lack of significant ADEs. This study represents an exploratory effort to determine if there is a role for sitagliptin therapy as adjunctive therapy for acute control of blood sugar management in the inpatient postoperative cardiac surgery setting after initial management with insulin protocols in the intensive care unit (ICU). 

## 2. Materials and Methods

### 2.1. Patients

The research protocol was approved by the institutional Investigational Review Board (IRB), and all patients provided informed consent. Patients greater than 18 years of age and who met all of the following criteria during the inpatient stay were eligible: T2DM controlled by diet or by oral medication at home; nonemergent cardiac surgery, including patients with aortic or mitral stenosis requiring valve replacement; ready to begin eating in the postoperative setting.

Patients were excluded from participation for any of the following reasons: emergency surgery, hemodynamic instability, pregnancy, type 1 diabetes mellitus (T1DM), T2DM which required insulin at home, males with a serum creatinine (SCr) concentration >3.0 mg/dL, females with a SCr concentration >2.5 mg/dL, total parenteral nutrition, enteral feeding, or patients on chronic oral steroids.

### 2.2. Study Design

This was a single-center, randomized, double-blind, placebo-controlled pilot study. Postoperative cardiac surgery patients were enrolled in the study once patients were transitioned from the ICU insulin protocol to a basal/prandial regimen (if necessary) and, ready to begin an oral diet. Patients were randomized using a computer-generated allocation schedule to receive either sitagliptin 100 mg tablet once daily for four days or a matching placebo for the same duration when they were ready to begin eating light solids. Both the active drug and matching placebo were compounded into matching capsules by a local compounding pharmacy. Males with a SCr concentration of 1.7 to ≤3.0 mg/dL or females with a SCr concentration >1.5 to ≤2.5 mg/L received 50 mg/day according to the package labeling [4]. The study medication was scheduled to begin on the same day the patient began eating solid food and transitioned to a basal/prandial regimen, if necessary.

All patients were administered the same carbohydrate-consistent (diabetic) diet and were instructed not to consume anything other than the diet provided by the site. Patients also received standard postoperative blood glucose protocol management following transfer from the intensive care unit, which consisted of a basal/prandial insulin, if warranted. Maintenance (home) of oral antihyperglycemic medications could be restarted when clinically indicated at the discretion of the physician. All postoperative blood glucose management decisions were managed by the physician and/or nurse practitioner who were blinded to the treatment group. 

### 2.3. Blood Glucose Measurements and Insulin Administration

Capillary blood glucose concentrations were obtained by nursing five times per day after study enrollment and continued throughout the study period. All blood glucose check times were standardized which occurred at 3:30 am, 7:30 am, 11:30 am, 4:30 pm, and 10 pm per protocol. The 3:30 am and 7:30 am were fasting measurements. A mean 24-hour blood glucose value was calculated for each 24-hour period after the study drug was initiated. 

If patients received any intravenous dextrose used as a diluent for medications or as continuous infusions, the amount of dextrose administered (in grams) was determined for each 24-hour period after study drug administration.

### 2.4. Study Endpoints and Definitions

The primary objective was to estimate the effect of adjunctive sitagliptin administration on overall mean blood glucose in the 4-day period after initiation of sitagliptin therapy compared to standard blood glucose management practices alone. A secondary objective was to estimate the effect of adjunctive sitagliptin on mean blood glucose control during each 24-hour period after initiation of sitagliptin therapy. Study day (SD) no. 1 was defined as the first 24-hour period after sitagliptin or placebo administration. Each subsequent 24-hour period after transfer was considered SD #2, SD #3, and SD #4. Mean capillary blood sugars measurements, from the time the study drug was initiated, were used to statistically compare the two study groups.

Safety and tolerability were assessed during the study. Monitoring for medication-related adverse effects, overall clinical status, and laboratory measurements (daily hematology and serum chemistries) were performed throughout the study. Patients were also monitored for occurrence of infection during the postoperative/study period. Occurrence of infection was defined as either radiographic or microbiologic evidence of infection from cultures or suspected infection with a temp >38°C or white blood count (WBC) >10,800 cells per cubic millimeter (cmm).

Episodes of hypoglycemia were of special interest during study drug administration. All patients were monitored for symptoms of hypoglycemia (per standard postoperative protocol orders) and treated for hypoglycemia per institutional protocol, if necessary. Hypoglycemia was defined as a blood glucose measurement <60 mg/dL and was identified as a discrete event once blood sugars return to normal, defined as >80 mg/dL. 

### 2.5. Statistical Analysis

Accurate prospective power analysis requires that the researchers have good information about the variables and sample from previous papers [9]. No literature is available about the role of sitagliptin in a hospital setting, making this an exploratory type of study. Therefore, we planned an enrollment goal based upon the cardiac surgery caseload in the past two years; we felt that 120 patients would be a reasonable sample size to attain.

Statistical analysis was conducted using SPSS version 18 (Statistical Package for Social Scientists). Participants were recruited and enrolled at the time of admission into this randomized repeated measures crossover study. The subjects received a sequence of different treatments, thereby reducing the influence of confounding covariates because each crossover patient served as his or her own control. Moreover, this design proved to be statistically effective, as it requires fewer subjects than noncrossover designs. *t* tests and longitudinal repeated measures for independent samples were used to compare the mean blood glucose levels for the overall 4-day study period as well as for each individual study day. The demographic variables of sex, height, and weight were compared between treatment groups using Pearson's chi-square statistic. The sitagliptin and placebo group effectiveness in controlling the blood glucose level was studied using the repeated measures analysis. 

## 3. Results

### 3.1. Baseline Assessment

Sixty-two patients were enrolled in the trial. Our original goal of 120 patients was not met due to a dramatic drop in the cardiac surgery caseload at this site and the exit of one surgeon during the study period. Thirty patients were assigned to the sitagliptin group, and 32 patients were assigned to the control group. Only one patient required dose adjusted for SCr, and this was required for only one dose of sitagliptin. Baseline analysis revealed no statistical differences between the treatment groups and control groups in demographics and baseline parameters (see Table 1). With respect to the sitagliptin and control groups, the mean patient ages were 62.9 and 66.1 years, respectively. Both groups were similar with respect to number of study participants of each sex at 21 and 27 males. Also, the groups were similar in that there was no statistically significant difference between weights with 93.1 kg and 94.2 kg for the treatment and placebo groups, respectively. There was no significant difference between the groups in various other baseline factors such as history of strokes, oral antihyperglycemic use, and type of procedures. 

### 3.2. Outcomes Assessment

The repeated measures test indicated that there was no significant difference between the groups in the overall mean blood glucose level with a mean of 147.2 ± 4.8 mg/dL and 153.0 ± 4.6 mg/dL for the test and the control group, respectively; *P* = 0.388 (Table 2). The blood glucose level did change significantly from day one to day four in both groups (*P* = 0.001). The result does not support sitagliptin as an adjunct therapy and thereby supports the null hypothesis. 

Further repeated measures analysis by controlling for oral antihyperglycemic use revealed that the mean blood glucose for the test and controlled group were 146.7 4.8 mg/dL and 153.0 4.6 mg/dL (*P* = 0.296), respectively, indicating that use of antihyperglycemic agents did not alter the results. A third step of repeated measures was performed by controlling for the oral antihyperglycemic and total insulin utilization. The differences in the mean blood glucose level for the test and control groups were further decreased to 145.5 4.2 mg/dL and 147.4 4.0 mg/dL, respectively (*P* = 0.778). The percentage of subjects with home medications restarted during study days 1–4 was evaluated and demonstrated no significant difference between treatment groups. These data are presented in Table 3.

Clinical endpoints such as incidence of postoperative infection requiring antibiotic therapy and length of postoperative hospital stay were also evaluated. Two patients in the sitagliptin group and four patients in the placebo group developed a postoperative infection requiring antibiotics (*P* = 0.675). The mean length of postoperative hospital stay was not statistically different between the sitagliptin and placebo groups at 6.4 ± 2.7 days and 7.5 ± 4.8 days, respectively; *P* = 0.276.

### 3.3. Safety Assessment

There were no reports of nausea, vomiting, or diarrhea among study patients. All patients were monitored for hypoglycemia. There were 7 discrete cases of hypoglycemia that occurred during the study; five episodes were in the sitagliptin group and two episodes in the placebo group (*P* = 0.055). 

## 4. Discussion

This exploratory study was performed to determine if sitagliptin added to standard postoperative blood glucose management improved mean overall blood glucose control during a four-day period after cardiac surgery compared to standard blood glucose practices alone. Negative circumstances arise from uncontrolled blood sugars in cardiac surgery patients, such as increased risk of bacterial infection, decreased tissue and organ perfusion, and compromised wound healing [10–12]. Tighter blood glucose control in the perioperative setting decreases the incidence of ischemic events, wound complications, and prolonging survival [13].

Sitagliptin has a novel mechanism of action that complements other antidiabetic therapies, a low risk of side effects, and a safe drug interaction profile [4–6]. However, despite these theoretical benefits, the results of this research demonstrated that adjunctive therapy with sitagliptin does not improve blood glucose control. 

Sitagliptin had no impact on overall mean blood glucose control over the entire 4-day study period. Furthermore, there were no significant differences between the two study groups for each 24-hour period from the time the study drug was initiated affirming that sitagliptin does not contribute significantly to blood glucose control. Concomitant dextrose and insulin requirements during the study period must be considered in order to properly evaluate this data. With the exception of only one day (SD #1), the mean number of grams of dextrose intake was similar between the groups. Additionally, the mean number of units of insulin was similar between the two groups throughout the entire study period. The groups were not controlled for insulin and dextrose utilization because mechanisms were necessary to ensure that patients would receive insulin for hyperglycemic events when required. 

Because blood glucose control can impact the incidence of infections in the postoperative patient, we evaluated the incidence of infection, antibiotic use, and postoperative length of stay in this study [10, 11]. Not surprisingly, clinical outcome parameters such as incidence of postoperative infection requiring antibiotics and the length of postoperative hospital stay were not different between groups. The authors would have expected that these parameters would only have been impacted if there had been a significant difference in blood glucose control between the two treatment groups. 

As part of the study protocol, the physician or physician assistant evaluated blood glucose control on a daily basis and added back home oral antihyperglycemic medications to the study drug (active drug or placebo) when they deemed it was clinically indicated. The percentage of study participants who were restarted on home oral antihyperglycemic agents increased over the course of the 4-day period. This increase occurred in both groups. However, this increase did not differ significantly between the two groups and was not unexpected as patients are eating more and feeling better. As the postoperative period lengthens, patients would be more likely to tolerate additional antihyperglycemic medications, and clinicians would be more comfortable starting these medications.

Safety endpoints were also evaluated in this research project. In a meta-analysis comparing sitagliptin to comparator oral medications, sitagliptin was less likely to cause hypoglycemia [5]. In our study, although not statistically significant, there was a strong trend demonstrating an increased likelihood for sitagliptin to cause hypoglycemia. In each case of hypoglycemia, this appears to be related to a recent dose of supplemental insulin the patient received (in both treatment groups) and not directly related to sitagliptin therapy. Although not all study participants required supplemental insulin therapy, this is the first research that documents experience with sitagliptin therapy used concomitantly with insulin therapy. 

There are a number of reasons why this pilot study did not show positive results such as those reported by Sokol et al. which were also conducted in a cardiac surgery population [7]. In the Sokol study, GLP-1 was administered as a continuous infusion before surgery and continued for 48 hours after surgery. The time frame for initiation of the study drug of the present study was different from Sokol study in that patients were not initiated on the study drug until the patient was ready to begin eating solid food and transitioned to a basal/prandial insulin regimen, if needed. Given that sitagliptin was administered orally once a day in the present study, and not by continuous infusion, it is possible that there was incomplete absorption of the medication and/or a lack of consistent blood concentrations of sitagliptin to prevent the breakdown of GLP-1. Absorption of oral medications can be hindered in postoperative patients due to the effects of narcotics and anesthesia on the gastrointestinal tract. Both of the studies allowed physicians, in a blinded fashion, to restart home antidiabetic medications. However, the Sokol study does not report the amount of insulin the patients receiving the GLP-1 infusions received. It is possible they received more insulin than patients reported in the present study, where overall insulin utilization was tracked and did not differ between the sitagliptin or placebo groups.

Other reasons may explain why the present study did not show the positive results compared to a previous paper in cardiac surgery patients [7]. When looking at the number of patients who had diabetes, only 3 patients in the Sokol study were diabetic, while all of the patients included in the present study had a diagnosis of T2DM. Thus, all of the patients will be less likely to produce adequate insulin in the face of lower endogenous insulin levels and higher levels of inflammatory markers, such as CRP, as a result of cardiac surgery [14]. 

There are several limitations to this research. Studying the effects of a diabetes drug for 4 days is problematic in that cumulative results over time may have influenced the nonsignificant results observed in this study. A small sample size limits the interpretation or generalizability. Another limitation is that the study population may be different from the typical clinical population. All patients received the same number of calories in their diabetic diet, but this does not mean that the patient ate all of each meal. Also, participating in a trial may influence the results due to an inherent selection bias; that is, patients who participate in clinical trials may somehow be nonrepresentative of the general clinical population of interest. Future research in this area could evaluate this medication used with insulin over a longer period of time as well as quantifying and reporting key plasma substances, such as GLP-1, GIP, and glucagon in combination with clinical outcomes in postoperative patients.

## 5. Conclusion

The addition of sitagliptin as an adjunct therapy to standard postoperative blood glucose management practices in diabetic patients does not affect the overall mean blood glucose concentrations in the postoperative setting. Sitagliptin therapy was well tolerated, with a strong trend towards causing more hypoglycemia compared to placebo.

## Figures and Tables

**Table 1:** Baseline patient profiles and operative characteristics.

Variable	Sitagliptin (*n* = 30)	Placebo (*n* = 32)	*P* value
Sex, male/female, *n*	21/8	27/5	0.255
Mean age, years	62.9 ± 9.6	66.1 ± 10.3	0.222
Height (inches)	67.5 ± 2.4	68.5 ± 3.6	0.241
Weight (kg)	93.1 ± 20.5	94.2 ± 18.0	0.822
Cardiovascular risk factors, *n* (%)			
History of stroke	3	1	0.255
History of CAD	1	0	
Oral antihyperglycemics			
Metformin, *n* (%)	48.1%	38.7%	0.469
Sulfonylureas, *n* (%)	25.9%	25.8%	0.992
Thiazolidinedione, *n* (%)	7.4%	16.1%	0.309
Preoperative A1c, %	7.21	7.27	0.879
Type of procedure, *n* (%)			0.282
CABG only	82.8%	84.4%	
CABG + valve	6.9%	0%	
Mean length of pump time, minutes	95.3 ± 30.9	88.4 ± 21.2	0.317
Mean length of ischemic time, minutes	67.2 ± 23.2	65.1 ± 17.9	0.707

**Table 2:** Study endpoints.

Endpoint	Sitagliptin (*n* = 30)	Placebo (*n* = 32)	*P* value
Mean blood glucose control (mg/dL)			
Overall 4-day study period	147.29 ± 4.79	153.0 ± 4.61	0.388
SD no. 1	160.5 ± 33.0	150.5 ± 28.5	
SD no. 2	163.1 ± 37.9	164.3 ± 33.9	
SD no. 3	137.4 ± 25.1	149.7 ± 30.6	
SD no. 4	128.2 ± 21.7	147.7 ± 34.5	

SD: study day.

**Table 3:** Antihyperglycemic medication utilization.

Variable	Sitagliptin (*n* = 30)	Placebo (*n* = 32)	*P* value
Overall insulin utilization			
Mean number of units over 4-day study period, *n*	13.2 ± 13.5	13.5 ± 12.6	0.942
Mean amount of dextrose utilized during study*, grams	4.8 ± 6.3	3.4 ± 3.4	0.307
Insulin utilization SD no. 1			
Mean number of units, *n*	32.5 ± 26.5	34.0 ± 36.5	0.857
Mean amount of dextrose, grams	13.7 ± 11.2	7.0 ± 7.5	0.008
Insulin utilization SD no. 2			
Mean number of units, *n*	10.1 ± 15.7	7.2 ± 11.5	0.411
Mean amount of dextrose, grams	1.4 ± 4.4	2.6 ± 7.0	0.437
Insulin utilization SD no. 3			
Mean number of units, *n*	6.3 ± 12.1	4.5 ± 8.3	0.500
Mean amount of dextrose, grams	1.5 ± 6.6	1.3 ± 4.1	0.917
Insulin utilization SD no. 4			
Mean number of units, *n*	5.7 ± 11.8	3.1 ± 5.2	0.319
Mean amount of dextrose, grams	1.4 ± 6.8	0.6 ± 1.6	0.593
Oral Agents			
Percentage of subjects with home medications restarted SD no. 1	53.3%	34.4%	0.212
Percentage of subjects with home medications restarted SD no. 2	70%	50%	0.179
Percentage of subjects with home medications restarted SD no. 3	70%	59.4%	0.543
Percentage of subjects with home medications restarted SD no. 4	70%	65.6%	0.923

*Amount of dextrose infused in medications and/or continuous infusions.

SD: study day.
